# Plasmatic Magnesium Deficiency in 101 Outpatients Living with Type 2 Diabetes Mellitus

**DOI:** 10.3390/clinpract11040095

**Published:** 2021-10-27

**Authors:** Hajer Zahra, Olfa Berriche, Ramla Mizouri, Fatma Boukhayatia, Marwa Khiari, Amel Gamoudi, Ines Lahmar, Nadia Ben Amor, Faten Mahjoub, Souheil Zayet, Henda Jamoussi

**Affiliations:** 1 Department of Nutrition, Metabolic Diseases and Diabetology, The National Institute of Nutrition and Food Technology of Tunis, Tunis 1007, Tunisia; lfaberriche1@gmail.com (O.B.); mizouriramla@gmail.com (R.M.); fatma.boukhayatia@gmail.com (F.B.); marwa.khiari92@gmail.com (M.K.); amel.gamoudi@gmail.com (A.G.); ines.lahmar@gmail.com (I.L.); nadia.benamor@gmail.com (N.B.A.); faten_mahjoub@yahoo.fr (F.M.); henda.jamoussi@gmail.com (H.J.); 2 Faculty of Medicine of Tunis, University Tunis El Manar, Tunis 1007, Tunisia; 3 Department of Infectious Diseases, Nord Franche-Comté Hospital, 90400 Trevenans, France

**Keywords:** type 2 diabetes, magnesium deficiency, glycemic control, diabetes complications

## Abstract

(1) Background: Magnesium deficiency is usually associated with type 2 diabetes mellitus (T2DM). Individuals living with T2DM with hypomagnesemia show a more rapid disease progression and have an increased risk for diabetes complications. (2) Methods: This is a cross-sectional and descriptive study in the National Institute of Nutrition and Food Technology of Tunis in Tunisia, including all adult outpatients (≥18 years old) with a diagnosis of T2DM from 1 September 2018 to 31 August 2019. The aim of this study was to evaluate the prevalence of plasmatic magnesium deficiency in a Tunisian population of T2DM and to study the relationship between magnesium status and intake, glycemic control and long-term diabetes-related complications. (3) Results: Among the 101 T2DM outpatients, 13 (12.9%) presented with a plasmatic magnesium deficiency. The mean age was 56 ± 7.9 years with a female predominance (62%, *n* = 63). The mean of the plasmatic magnesium level was 0.79 ± 0.11 mmol/L (0.5–1.1), and the mean of 24 h urinary magnesium excretion was 87.8 ± 53.8 mg/24 h [4.8–486.2]. HbA1c was significantly higher in the plasmatic magnesium deficiency group than the normal magnesium status group (10% ± 1.3 vs. 8.3% ± 1.9; *p* = 0.04), with a significant difference in participants with a poor glycemic control (HbA1c > 7%) (100%, *n* = 13/13 vs. 53%, *n* = 47/88; *p* = 0.001). A weak negative relationship was also found between plasmatic magnesium and HbA1c (r = −0.2, *p* = 0.03). Peripheral artery disease was more commonly described in individuals with low plasmatic magnesium levels than in individuals with normal levels (39%, *n* = 5 vs. 0%, *n* = 0; *p* < 0.001). The mean plasmatic magnesium level in participants without diabetic nephropathy and also peripheral artery disease was significantly higher compared to individuals with each long-term diabetes-related complication (0.8 mmol/L ± 0.1 vs. 0.71 mmol/L ± 0.07; *p* = 0.006) and (0.8 mmol/L ± 0.1 vs. 0.6 mmol/L ± 0.08; *p* < 0.001), respectively. (4) Conclusions: Hypomagnesemia was identified in individuals with T2DM, causing poor glycemic control and contributing to the development and progression of diabetes-related microvascular and macrovascular complications.

## 1. Introduction

Magnesium is considered to be the second most important intracellular cation after potassium. It is implicated in more than 300 metabolic reactions in the body, and its deficiency causes biochemical disorders and alterations in the human organism [1]. It serves as an important cofactor in many essential enzymatic reactions, and it is involved in glucose metabolism and several physiological processes, including modulation of smooth muscle tone and endothelial cell function [2]. Magnesium is widely distributed in plant and animal foods and in beverages. Foods containing dietary fiber and green leafy vegetables, such as spinach, legumes, nuts, seeds, and whole grains, are good sources to provide magnesium [3]. The recommended dietary allowance for magnesium for adult men is 400–420 mg per day for adult men and 310–320 mg per day for adult women [3]. The U.S. Department of Agriculture has listed the content of many foods and has provided comprehensive list of foods containing magnesium arranged by nutrient content and by food name [4]. This mineral could interact with different pathophysiological mechanisms, including insulin secretion, insulin resistance and parameters of glucose homeostasis [2,5].

Consistent epidemiological data have established a relationship between plasmatic magnesium deficiency or hypomagnesemia and the increased prevalence of type 2 diabetes mellitus (T2DM) [6,7,8]. Emerging epidemiological evidence has supported that magnesium intake is significantly inversely associated with risk of T2DM [9], and a statistically significant linear dose-response relationship was established between incremental magnesium intake and T2DM risk [10]. Indeed, magnesium deficiency is involved in glucose intolerance and insulin resistance [7,8,11], and could be associated with poor glycemic control. Magnesium deficiency could also be a risk factor for the development or progression of long-term diabetes related complications.

Until now, the mechanism inducing magnesium deficiency in diabetes has been unclear. It is now established that diabetes can induce hypomagnesemia and that hypomagnesemia can in turn induce or deteriorate this disease [7]. In addition, several studies have shown that magnesium supplementation has a beneficial effect on insulin action and glucose metabolism [12,13]. To our best knowledge, this is the first reported study in the African continent to estimate the prevalence of magnesium deficiency in a population of Tunisian outpatients with T2DM and to evaluate the association between magnesium status and intake, glycemic control and diabetes microvascular and macrovascular complications. The aim of this study was (i) to determine the prevalence of plasmatic magnesium deficiency in T2DM participants and describe the clinical and biological characteristics of this population, (ii) to compare two groups of individuals: those living with diabetes with normal serum magnesium levels and those with plasmatic magnesium deficiency, and, finally, (iii) to determine the relationship between magnesium status and intake, glycemic control and the occurrence of microvascular and macrovascular complications of T2DM.

## 2. Materials and Methods

### 2.1. Study Design

The present study was a cross-sectional and descriptive study undertaken at the National Institute of Nutrition and Food Technology of Tunis in Tunisia.

### 2.2. Participants

We included all adult outpatients (≥18 years old) with a diagnosis of T2DM and treated with oral anti-diabetic drugs and/or insulin during a one-year period from 1 September 2018 to 31 August 2019. We formally excluded pregnant and lactating women, participants treated with agents or drugs that may interfere with magnesium metabolism (proton pump inhibitors, cisplatin, diuretics, antibiotics such as tetracyclines and quinolones, oral contraceptives) or who received oral magnesium supplementation recently, and participants with a chronic past history of alcoholism, chronic diarrhea (Crohn’s or celiac disease), kidney failure (creatinine clearance ˂ 60 mL/min according to the CKD-EPI), hepatic and/or heart failure. We also excluded from this study any individual declining to participate or expressing an opposition to data collection from hospital information systems.

### 2.3. Baseline Characteristics

Clinical data regarding demographic and baseline characteristics and diabetes history were collected through our participants’ interrogation and medical records. Participants’ data collection form was elaborated and approved by our experts (Supplementary Materials). A 24 h food recall was used to determine magnesium intake and was conducted by trained nutritionists who employed a method of the food history which specifically guided participants in evaluating the proportions of food consumed at each meal. The foods were translated into nutrients by referring to the food composition tables proposed in the National Institute of Nutrition and Food Technology of Tunis in Tunisia [14]. The exploitation of the results of this survey was analyzed using the BILNUT Software (Version 4.0, Nutrisoft, Cerelles, France) On-line Computerized System, to which lists of 235 special Tunisian foods were added (Dietary fiber in the diets of urban Tunisian women: Association of fiber intake with BMI, waist circumference and blood chemistry: Preliminary study) in order to estimate magnesium intake.

We also collected information regarding microvascular and macrovascular diabetes complications through the medical record and biological findings, including fasting blood glucose, glycated haemoglobin (HbA1c), plasmatic magnesium, 24 h urinary magnesium excretion, serum lipid profile (total cholesterol, HDL cholesterol, LDL cholesterol and triglycerides) and serum creatinine clearance. Microvascular diabetes complications were defined as diabetic retinopathy, neuropathy and nephropathy, whereas macrovascular complications were defined as coronary heart disease, cerebrovascular disease and peripheral artery disease.

### 2.4. Plasmatic and Urinary Magnesium Levels Determination

The magnesium determination was based on the reaction of magnesium with Xylidyl Blue-I (as chelator) at alkaline pH 11.4, which yields a purple-colored complex. The color produced is measured bichromatically at 520/800 nm and is proportional to the magnesium concentration. We considered a low level of magnesium (hypomagnesemia) if plasmatic magnesium concentration < 0.65 mmol/L [15]. Concerning 24 h magnesuria, urinary magnesium was collected and determinate by the colorimetric method. The 24 h urine was diluted 1:10 with distilled water before the assessment. Normal levels of magnesium in 24 h urine were between 60 and 120 mg/24 h, according to threshold values of our laboratory.

### 2.5. Data Analysis

Continuous variables were expressed as mean and standard deviation (SD) and compared with Student’s t-test. Categorical variables were expressed as a number (%), and compared by a Chi-square test or Fisher’s exact test between the two groups (participants with normal magnesium status and participants with plasmatic magnesium deficiency). Relationships between two quantitative variables were studied by Pearson’s correlation coefficient, and in case of non-validity, by Spearman’s rank correlation coefficient. A *p*-value < 0.05 was considered significant. We used SPSS v24.0 software (IBM, Armonk, NY, USA).

### 2.6. Ethics Statement

Due to the non-prospective nature of the study, the Ethics Committee of the National Institute of Nutrition and Food Technology of Tunis in Tunisia determined that participant consent was not required. The confidentiality of participant data has been respected in accordance with the Declaration of Helsinki.

## 3. Results

During the study period, 101 outpatients living with T2DM were included in our facility. Of those participants, 88 (87%) were in the normal magnesium status group, and 13 (13%) were in the plasmatic magnesium deficiency group (Table 1). The mean age was 56 ± 7.9 years with a female predominance (62%, *n* = 63). The mean plasmatic magnesium level was 0.8 ± 0.1 mmol/L (0.5–1.1) and the mean 24 h urinary magnesium excretion was 87.8 ± 53.8 mg/24 h (4.8–486.2), with a significant relationship between the two items (r = 0.21; *p* = 0.04). The mean diabetes duration in the whole population was 7.2 ± 5.4 years (1–25). Plasmatic serum magnesemia decreased proportionally with the diabetes duration with no significant relationship (r = 0.12; *p* = 0.22).

### 3.1. Description of Participants with Plasmatic Magnesium Deficiency and Comparison of the Two Groups

In participants with plasmatic magnesium deficiency (*n* = 13), the mean age was 54 ± 5.8 years (45–66), and 11 (85%) of those participants were female. More than two-thirds of participants (69%, *n* = 9) were between 51 and 65 years. The mean body mass index (BMI) was 29 ± 2.9 (23.5–33.6). Furthermore, the mean diabetes duration in participants with plasmatic magnesium deficiency was 7.62 ± 3.9 years (1–15). Dietary magnesium or intake was estimated to be about 274.7 ± 105.8 mg/d (148–367). 24 h urinary magnesium excretion was lower in participants with plasmatic magnesium deficiency (56.4 ± 38 mg/24 h) compared to those with normal status (93 ± 54.5 mg/24 h), with a significant difference (*p* = 0.02).

The main microvascular complications were diabetic retinopathy and neuropathy (39%; *n* = 5 for each one). Moreover, peripheral artery disease was the most common macrovascular complication observed (39%, *n* = 5). Concerning biological findings, the mean glucose level was 11.2 ± 2.9 mmol/L (6.4–15.4) and the mean HbA1c was 10 ± 1.3% (8.3–12.6). All participants had HbA1c > 7%, which implies poor glycemic control. Laboratory renal and lipid parameters were detailed in Table 1. Among the 101 participants, HbA1c was significantly higher in the plasmatic magnesium deficiency group than the normal magnesium status group (10% ± 1.3 vs. 8.3% ± 1.9; *p* = 0.04). No significant difference was found regarding BMI and diabetes duration. A significant difference was found in individuals with a poor glycemic control (HbA1c > 7%) (100%, *n* = 13/13 vs. 53%, *n* = 47/88; *p* = 0.001). Concerning long-term diabetes-related complications, it is important to emphasize that peripheral artery disease was only present in participants with low plasmatic magnesium level and not in participants with normal magnesium levels (39%, *n* = 5 vs. 0%, *n* = 0; *p* < 0.001).

### 3.2. Association between Magnesium Status and Glycemic Control

**Figure 1:**
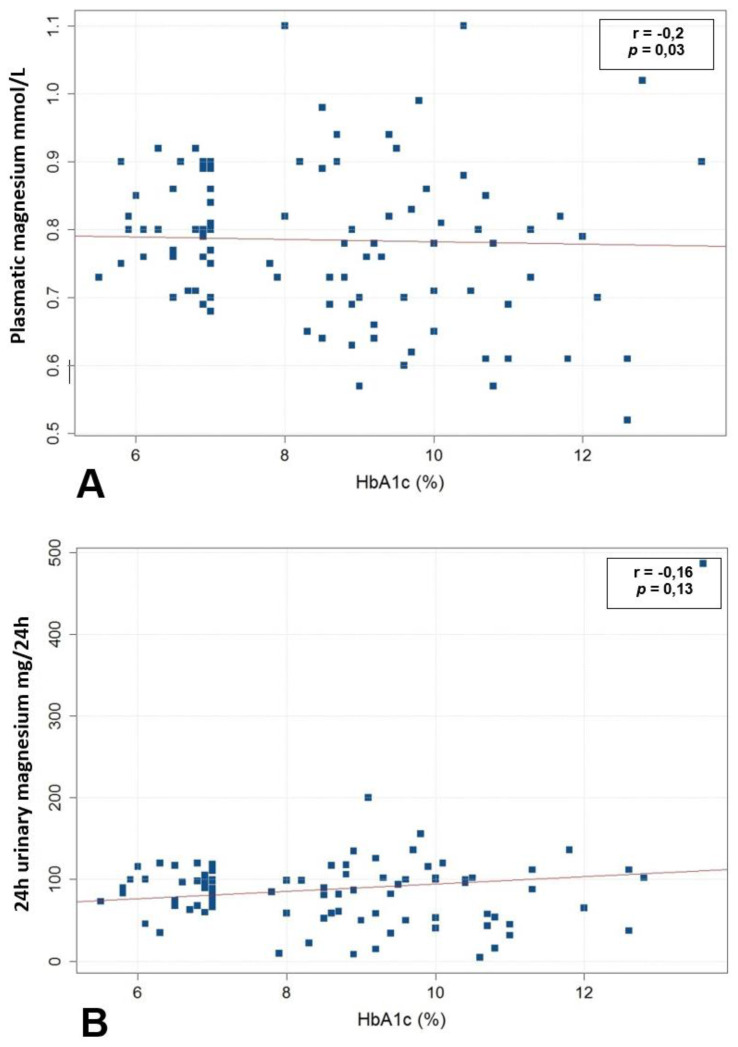
(**A**) Relationship between plasmatic magnesium concentrations and HbA1c in all participants living with type 2 diabetes. (**B**) Relationship between 24 h urinary magnesium concentrations and HbA1c in all participants living with type 2 diabetes (*n* = 101).

A weak negative relationship was found between plasmatic magnesium and HbA1c (r = −0.2, *p* = 0.03) (Figure 1A). Plasmatic magnesium was significantly higher in participants with well-controlled diabetes (HbA1c < 7%) than in participants with poor glycemic control (HbA1c > 7%) (0.84 mmol/L vs. 0.76 mmol/L, *p* = 0.03). However, concerning the nutritional profile, dietary magnesium per day was lower in individuals with poor glycemic control than in individuals with well-controlled diabetes (303 mg/d vs. 363.7 mg/d, *p* = 0.2), with no significant difference. Finally, 24 h urinary magnesium increased proportionally with HbA1c, but the relationship was not statistically significant (r = 0.16, *p* = 0.13) (Figure 1B).

### 3.3. Association between Magnesium Status and Long-Term Diabetes-Related Complications

The mean plasmatic magnesium level in individuals without diabetic nephropathy was significantly higher compared to individuals with diabetic nephropathy (0.79 mmol/L ± 0.1 vs. 0.7 mmol/L ± 0.07; *p* = 0.006). The mean plasmatic magnesium level in individuals without peripheral artery disease was higher than in individuals with peripheral artery disease, with a significant difference observed (0.8 mmol/L ± 0.1 vs. 0.6 mmol/L ± 0.08; *p* < 0.001). Table 2 describes plasmatic magnesium levels according to the presence or absence of diabetic microvacular and macrovascular complications.

## 4. Discussion

In this study including 101 T2DM outpatients, 12.9% presented with a plasmatic magnesium deficiency. HbA1c was significantly higher in the plasmatic magnesium deficiency group than in the normal magnesium status group (*p* = 0.04), with a significant difference in participants with poor glycemic control (HbA1c > 7%) (*p* = 0.001). A weak relationship was also found between plasmatic magnesium and HbA1c (r = −0.2, *p* = 0.03). The mean plasmatic magnesium level in participants without diabetic nephropathy and peripheral artery disease was significantly higher compared to individuals with each long-term diabetes-related complication (*p* = 0.006 and *p* < 0.001, respectively).

Magnesium deficiency is usually associated with T2DM, increased insulin resistance and reduced insulin secretion [16], thus contributing to uncontrolled diabetes. Several mechanisms have been suggested to explain the correlation between magnesium deficiency and T2DM. Indeed, magnesium may act by (i) enhancing insulin secretion through a direct or indirect effect mediated by intracellular calcium [17], (ii) inhibiting the inositol 1,4,5-triphosphate (IP3)-gated calcium channel—thus magnesium may be acting as a calcium antagonist, (iii) reducing peripheral insulin resistance by stimulating tyrosine kinase activity, a component of the beta subunit of the insulin receptor for which magnesium is a cofactor [18,19], (iiii) activating acetyl-CoA carboxylase, which catalyzes the formation of long-chain fatty acids and plays a role in insulin secretion [20] and (iiiii) playing a role in the regulation of genomic transcription by increasing pancreatic GLUT2 and insulin mRNA expression [17]. Therefore, magnesium deficiency would lead to post-receptor insulin resistance development and contribute to decreased cellular glucose use [21]. This is due to disturbances of tyrosine kinase activity on the insulin receptor and glucose transport alteration.

In medical literature, the prevalence of hypomagnesemia in individuals living with T2DM is about 11–47% in almost all studies and case series [22,23,24,25,26,27,28], which is higher than our prevalence. This prevalence is particularly higher in African countries, where the nutritional intake of magnesium is probably insufficient [26,27,28]. This large difference in these results is related to the differences in magnesium measurement and monitoring methods in heterogeneous populations with different population sizes. Data from Meyer et al. [29] supported a protective role for dietary magnesium (more than 332 mg/d) in the development of diabetes in older menopausal women. In terms of gender difference, it is interesting to note that independent studies have reported a higher incidence of hypomagnesemia in women compared with men [7,30,31], which is also seen in our results. This can also be explained by female predominance in our participants living with T2DM. In our study, a significant relationship (r = 0.21; *p* = 0.04) was observed between 24 h urinary magnesium excretion and plasmatic magnesium level. This suggests that urinary magnesium excretion would decrease in case of hypomagnesemia.

Concerning diabetes duration, our results are similar to literature data where authors concluded that there is no relationship between low magnesium levels and T2DM duration [6]. In our study, HbA1c was significantly higher in the plasmatic magnesium deficiency group than the normal magnesium status group (*p* = 0.04). These results are consistent with those of Odusan et al. [26] and Fujii et al. [32]; these studies suggested a strong relationship between glycemic control and plasmatic magnesium deficiency. However, Walti et al. [8] concluded that plasmatic magnesium was not correlated with glycemic control as measured by HbA1c. Magnesium deficiency also plays an important role in the development and progression of diabetes-related microvascular and macrovascular complications. In our cohort, the mean plasmatic magnesium level in individuals without diabetic nephropathy or peripheral artery disease was significantly higher compared to individuals with long-term diabetes-related complications. The prevalence of macrovascular complications and peripheral artery disease in Tunisian people with diabetes has been previously estimated to be around 14.9% and 5%, respectively. This is likely underestimated by the asymptomatic nature of less severe peripheral artery disease [33,34,35] and the limited screening strategies and follow-up available in low-middle income countries.

In the observational Atherosclerosis Risk in Communities study including more than 13,000 participants, low serum magnesium has been associated with kidney function decline in individuals with diabetes [36]. In our study, the mean plasmatic magnesium level in individuals with diabetic nephropathy was significantly lower compared to individuals without diabetic nephropathy (*p* = 0.006). Sharma et al. [37] observed a strong association between hypomagnesaemia and diabetic retinopathy and concluded that serum magnesium level may be used as an early predictor of course and complications of diabetes mellitus. The mechanism underlying the hypomagnesemia and vascular complications association may be explained by magnesium vascular and endothelial function [38]. Magnesium deficiency may also contribute to the progression of atherosclerosis through its effect on lipid metabolism [39,40], platelet aggregation and blood pressure control [6,41].

Finally, we think that our data are relevant as the first Tunisian study that evaluates magnesium status in individuals with T2DM, which may help our healthcare systems in the management of this complex disease. However, there are a few limitations of this study: (i) the number of participants is limited, especially in the various subgroups (i.e., peripheral artery disease), to draw definitive conclusions, although our data is significant; (ii) this study is observational and thus cannot provide evidence of a causal relationship between magnesium and diabetes adverse outcomes, given the lack of a control group; (iii) pharmacological treatment of this population has not been collected and, therefore, the analysis has not been adjusted for this variable, which can be a determining factor; (iv) sequential erythrocyte magnesium was not monitored in our facility and so not monitored in magnesium intake and status.

## 5. Conclusions

This study confirms that magnesium deficiency was found in individuals with T2DM, estimated at about 13% of participants, especially in females. This may cause poor glycemic control and contribute to the development and progression of diabetes-related health complications. Indeed, in T2DM individuals with hypomagnesemia, HbA1c can be higher with more present peripheral artery disease compared to those with normal plasmatic levels. A hypomagnesemic state was reported to be associated with various micro- and macrovascular complications, such as diabetic nephropathy and peripheral artery disease. All these conclusions should lead physicians and diabetologists to systematically screen for plasmatic and urinary magnesium deficiency and support magnesium supplementation in individuals living with T2DM, and should help the overall management of this complex disease.

## Figures and Tables

**Table 1:** Comparative table between participants with normal magnesium status and participants with plasmatic magnesium deficiency in a population with T2DM, in the National Institute of Nutrition and Food Technology of Tunis in Tunisia (*n* = 101).

		Participants with Normal Magnesium Status (*n* = 88) (87.1%)	Participants with Plasmatic Magnesium Deficiency (*n* = 13) (12.9%)	*n* = 101 (%)	*p*-Value
Demographic and Baseline Characteristics					
Age, years (mean, SD, range)		56.3 ± 8.2 (39–76)	54.4 ± 5.8 (45–66)	56 ± 7.9 (39–76)	0.421
Sex (*n*, %)	Male	36 (40.9)	2 (15.4)	38 (37.7)	0.067
	Female	52 (59.1)	11 (84.6)	63 (62.4)	0.067
BMI (kg/m^2^) (mean, SD, range) Menopause (*n*, %)		30 ± 4.6 (19.6–45.7) 36/52 (69.2)	29 ± 2.9 (23.5–33.6) 9/11 (81.8)	29.9 ± 4.5 (19.6–45.7) 45/63 (71.4)	0.483 0.132
Diabetes duration, years (mean, SD, range)		7.11 ± 5.6 (1–25)	7.62 ± 3.9 (1–15)	7.18 ± 5.4 (1–25)	0.757
	=<10 (*n*, %)	72 (81.8)	11 (84.6)	83 (82.2)	0.581
	(11–20) (*n*, %)	14 (15.9)	2 (15.4)	16 (15.8)	0.662
	>20 (*n*, %)	2 (2.3)	0	2 (2)	0.758
Microvascular complications (*n*, %)	Diabetic retinopathy	22 (26.8)	5 (38.5)	27 (26.7)	0.289
	Diabetic neuropathy	16 (21.6)	5 (38.5)	21 (20.8)	0.129
	Diabetic nephropathy	12 (14.8)	2 (15.4)	14 (13.7)	0.615
Macrovascular complications (*n*, %)	Coronary heart disease	9 (10.2)	1 (7.7)	10 (9.9)	0.636
	Cerebrovascular disease *	6 (6.8)	2 (15.4)	8 (7.9)	0.261
	**Peripheral artery disease**	**0 (0)**	**5 (38.5)**	**5 (4.9)**	**<0.001**
**Plasmatic magnesium level (mmol/L)(mean, SD, range)** **24 h urinary magnesium excretion (mg/24 h)(mean, SD, range)** Magnesium intake ** (mg/24 h) (mean, SD, range)		**0.8 ± 0.1 (0.68–1.1)** **93 ± 54.5 (4.8–486.2)** 324.5 ± 111.8 (167–625)	**0.6 ± 0.3 (0.5–0.6)** **56.4 ± 38 (8.6–136)** 274.7 ± 105.8 (148–367)	**0.8 ± 0.1 (0.5–1.1)** **87.8 ± 53.8 (4.8–486.2)** 320.4 ± 111.2 (148–625)	**<0.001** **0.02** 0.397
Fasting blood glucose (mmo/L) (mean, SD, range)		9.8 ± 3.2 (4.3–19)	11.2 ± 2.9 (6.4–15.4)	10 ± 3.2 (4.3–19)	0.145
**HbA1c (%) (mean, SD, range)**		**8.3 ± 1.9 (5.5–13.6)**	**10 ± 1.3 (8.3–12.6)**	**8.5 ± 1.9 (5.5–13.6)**	**0.03**
**HbA1c > 7%**		**47 (53.4)**	**13 (100)**	**60 (59.4)**	**0.001**
Clearance creatinine *** (>60 mmo/L) (mean, SD, range)		99.8 ± 12.7 (61–140)	101.2 ± 12 (70–121)	99.9 ± 12.5 (61–140)	0.698
Total cholesterol (normal range < 5.2 mmol/L (mean, SD, range)		4.5 ± 0.9 (2.2–7)	4.3 ± 0.8 (3.4–6.5)	4.50 ± 0.9 (2.24–7.08)	0.565
HDL-c (normal range > 1 mmol/L (M) and >1.3 mmol/L (F)) (mean, SD, range)		1.1 ± 0.3 (0.5–1.8)	1.2 ± 0.2 (1–1.6)	1.16 ± 0.8 (0.51–1.80)	0.325
LDL-c **** (normal range < 2.6 mmol/L) (mean, SD, range)		1 ± 0.3 (0.3–2)	0.9 ± 0.3 (0.5–1.6)	1.02 ± 0.3 (0.35–2.03)	0.242
Triglycerides (normal range < 1.7 mmol/L) (mean, SD, range)		1.5 ± 0.8 (0.5–5.9)	1.5 ± 0.7 (0.8–3.3)	1.49 ± 0.8 (0.47–5.89)	0.828

Bold: significant difference (*p* < 0.05). Abbreviations (alphabetic order): HbA1c: glycated haemoglobin; HDL-c: HDL cholesterol; LDL-c: LDL cholesterol; SD: standard derivation. * Cerebrovascular diseases are defined as acute cerebral stroke and transient ischemic attack; ** we considered a normal daily magnesium intake mean as 420 mg/24 h (M) and 360 mg/24 h (F) according to AFSSA 2001 recommendations; *** we used using the CKD-EPI creatinine equation to estimate glomerular filtration rate (GFR), as per the National Kidney Foundation recommendation; **** we used Friedewald’s formula for the estimation of LDL-C concentration: LDL-c (mmol/L) = Total cholesterol − HDL-c − Triglycerides/2.2.

**Table 2:** Plasmatic magnesium level according to the presence or absence of microvacular and macrovascular complications in the National Institute of Nutrition and Food Technology of Tunis in Tunisia (*n* = 101).

	Diabetic Microvascular and Macrovascular Complications	Yes/No	Plasmatic Magnesium Level (mmol/L) (Mean, SD)	*p-*(Value)
Microvascular complications	Diabetic retinopathy	Yes	0.76 ± 0.12	0.3
		No	0.78 ± 0.10	
	Diabetic neuropathy	Yes	0.73 ± 0.13	0.1
		No	0.78 ± 0.10	
	**Diabetic nephropathy**	**Yes**	**0.71 ± 0.07**	**0.006**
		**No**	**0.79 ± 0.10**	
Macrovascular complications	Coronary heart disease	Yes	0.77 ± 0.10	0.63
		No	0.79 ± 0.09	
	Cerebrovascular disease	Yes	0.74 ± 0.12	0.31
		No	0.78 ± 0.13	
	**Peripheral artery disease**	**Yes**	**0.61 ± 0.08**	**<0.001**
		**No**	**0.79 ± 0.1**	

Bold: significant difference (*p* < 0.05). Abbreviations: SD: standard derivation.

## Data Availability

Data are available on request due privacy to restrictions. The data presented in this case study are available on request from the corresponding author.

## Supplementary Materials

The following is available online at https://www.mdpi.com/article/10.3390/clinpract11040095/s1, Participant’s living with type 2 diabetes data collection form.
