# Supplementary material for: Plasmatic Magnesium Deficiency in 101 Outpatients Living with Type 2 Diabetes Mellitus
Source: Clin Pract. 2021 Oct 27;11(4):791–800. doi: 10.3390/clinpract11040095 (PMC8628662; doi:10.3390/clinpract11040095)
Supplement: Supplementary file 1 [file clinpract-11-00095-s001.zip › clinpract-1410014-supplementary.pdf]

Participant's living with type 2 diabetes data collection form

The *National Institute of Nutrition and Food Technology of Tunis* in Tunisia

Family Name:…………….:…………….. ID number:……………… Address:……………….

Tel number: ………………..

Age:…………… Sex: F ☐ M ☐ Menopause (for F) : yes ☐ no ☐

Past history: Arterial hypertension: yes ☐ no ☐

Dyslipidemia: yes ☐ no ☐ : type:

Others:…………………

Clinical examination:

Blood pressure:…/…..mmHg Heart beats:……… bpm

	Weight (kg)	Height (m)	BMI (kg/m^2)
T0			

Diabetes:

Diabetes duration: ……….(years)

Diabetes Complications:

❖ Microvascular:

- ✓ Diabetic retinopathy: yes ☐ no ☐
- Diabetic nephropathy: yes ☐ no ☐
- ✓ Diabetic neuropathy: yes ☐ no ☐

❖ Macrovascular:

- ✓ Coronary heart disease: yes ☐ no ☐
- ✓ Cerebrovascular diseases: yes ☐ no ☐ type : Acute cerebral stroke ☐

Transient ischemic attack ☐

- ✓ Peripheral artery disease: yes ☐ no ☐

Biological Data:

**Fasting blood glucose (mmol/l)**	
**HbA1c (%)**	
**Plasmatic magnesium level (mmol/l)**	
**24h magnesium (mmol/l)**	
**Magnesium intake (mg/24h)**	
**Total cholesterol (mmol/l)**	
**HDL-c (mmol/l)**	
**LDL-c (mmol/l)**	
**Triglycerides (mmol/l)**	
**Clearance creatinine (CKD-EPI) (mmol/l)**	
